# Fibroma of tendon sheath extending to the radiocarpal joint with median nerve compression: A case report

**DOI:** 10.1097/MD.0000000000043735

**Published:** 2025-08-01

**Authors:** Nhat Tien Tran, Thuy Tram Ngo, Bao Song Nguyen Tran, Hong Phuc Le, Nghi Thanh Nhan Le

**Affiliations:** a Department of Surgery, University of Medicine and Pharmacy, Hue University, Hue, Vietnam; b Department of Orthopaedic & Plastic Reconstructive Surgery, Hue University of Medicine and Pharmacy Hospital, Hue, Vietnam; c Department of Histology, Embryology, Pathology and Forensic, University of Medicine and Pharmacy, Hue University, Hue, Vietnam.

**Keywords:** Fibroma of tendon sheath, median nerve compression, radiocarpal joint

## Abstract

**Rationale:**

Fibroma of tendon sheath (FTS) is a rare benign tumor arising from the synovial membrane of the tendon sheath. It seldom causes peripheral nerve compression or extends into a synovial joint. We report an unusual case of FTS within the radiocarpal joint, compressing the median nerve and leading to secondary changes in the radius and scapholunate joint.

**Patient concerns:**

A 59-year-old woman presented with a 2-year history of a progressively enlarging, tender mass on the volar aspect of her right wrist. She exhibited positive Tinel’s sign and Phalen’s test. Magnetic resonance imaging revealed a hypointense mass on T1-weighted images and a heterogeneously hyperintense mass on T2-weighted images, extending into the radiocarpal joint.

**Diagnoses:**

The patient was diagnosed with FTS based on histopathological analysis.

**Interventions:**

Surgical excision of the tumor was performed, along with decompression of the median nerve.

**Outcomes:**

At the 6-month follow-up, the patient had complete resolution of symptoms with no signs of recurrence.

**Lessons:**

FTS should be considered in the differential diagnosis of soft tissue masses causing nerve compression. Magnetic resonance imaging plays a crucial role in early detection and preoperative planning, especially in identifying intra-articular extension. However, histopathological examination remains the gold standard for definitive diagnosis. Complete surgical excision is the primary treatment, requiring meticulous preservation of joint structures and nerve function to minimize recurrence and optimize outcomes.

## 1. Introduction

Fibroma of tendon sheath (FTS) is a rare benign tumor arising from the synovial membrane of the tendon sheath. First fully described by Chung et al in 1979 in a series of 138 cases, this remains the most extensive study on FTS. The tumor most commonly occurs in adults aged 20 to 50, with a male predominance (75%). It frequently appears in the fingers (49%), hands (21%), wrists (12%), and knees (5%), with the flexor surfaces of the digits and palm being the most common locations.^[1]^ The precise pathogenesis of FTS is unknown, though some theories suggest prior trauma or mechanical stress as potential contributing factors. It is thought to be a reactive process rather than a true neoplasm.^[2]^ Histologically, FTS is characterized by low cellularity, spindle-shaped cells within a collagenous background, and slit-like spaces within the fibrous tissue.^[1]^ A definitive diagnosis requires clinical assessment, imaging, and histopathological confirmation. Due to its similarities with giant cell tumor of tendon sheath (GCTTS), distinguishing FTS from other soft tissue tumors can be challenging. FTS typically presents as a slow-growing, firm, and painless mass. Symptoms are uncommon unless the tumor compresses nearby structures. FTS is a rare cause of carpal tunnel syndrome.^[3]^ Intra-articular involvement and bony abnormalities are even rarer. This report presents a sporadic case of FTS arising from the radiocarpal joint, compressing the median nerve, and affecting the radius and scapholunate, highlighting the importance of early diagnosis and appropriate surgical management.

Written informed consent has been obtained from the patient to publish this case report. Ethical approval for this study was waived by the ethics committee of the Hue University of Medicine and Pharmacy Hospital because it was a case report.

## 2. Case presentation

**Figure 1.**
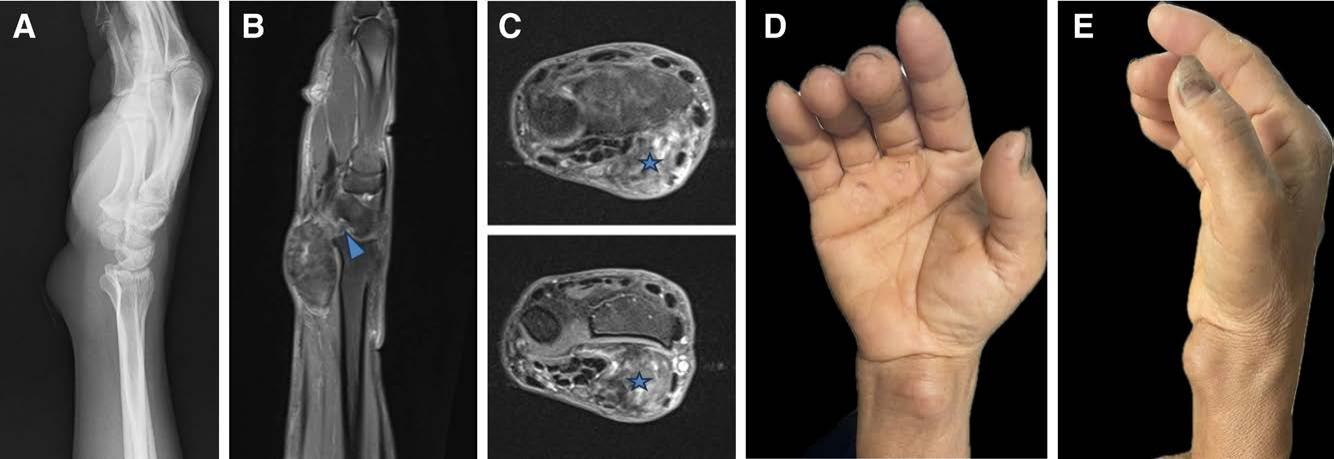
Preoperative clinical and imaging examination. (A) Lateral radiograph of the wrist showing subtle scalloping of the volar aspect of the distal radius. (B) Sagittal T2/PD-weighted MRI demonstrating a heterogeneous and slightly hypointense mass at the wrist, extending into the radiocarpal joint (arrowhead). (C) The tumor (star) displaces adjacent structures medially. (D, E) Anteroposterior and lateral views of the tumor. MRI = magnetic resonance imaging, PD = proton density.

A 59-year-old Vietnamese housewife presented with a 2-year history of a progressively enlarging, tender mass on the volar aspect of her right wrist. She reported numbness, tingling in the radial 3 fingers, and mild thumb weakness. There was no history of trauma in this area. Examination revealed a firm, tender mass, with positive Tinel’s sign and Phalen’s test. Her preoperative visual analog scale score was 7, and her QuickDASH score was 59.^[4]^ X-ray imaging of the right wrist revealed no calcifications or direct bony involvement, but the distal radius appeared concave due to compression by the mass. Magnetic resonance imaging (MRI) demonstrated a well-defined, multilobulated mass displacing the flexor tendons, compressing the median nerve, and extending into the radiocarpal joint. The lesion appeared hypointense on T1-weighted images and heterogeneously hyperintense on T2-weighted images, consistent with a fibrous tumor (Fig. 1). The primary diagnostic challenge was distinguishing between a soft tissue tumor compressing the median nerve and a tumor originating from the nerve itself.

**Figure 2.**
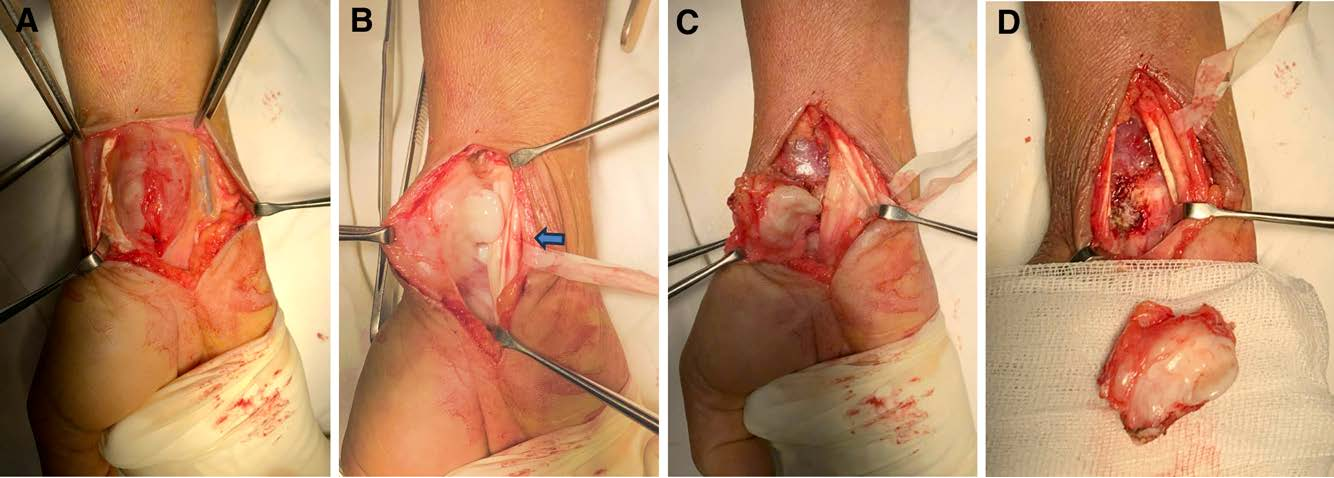
Intraoperative images. (A) The mass was densely adhered to the distal tendon of the wrist. (B) After opening the sheath, the wrist flexor tendons were exposed, revealing that the tumor was not directly attached to the median nerve (arrow). (C) The tumor involves the radiocarpal joint. (D) Joint capsule incision following complete tumor removal (arrowhead).

The patient then underwent surgical excision under brachial plexus anesthesia with tourniquet control. A volar wrist approach exposed a 4 cm × 3 cm × 2.5 cm multilobulated, firm mass proximal to the carpal tunnel. The flexor tendons and median nerve were ulnarly displaced, but there was no direct tumor attachment to the nerve. Due to its communication with the radiocarpal joint, a small capsular incision was made for complete excision. The tumor was carefully dissected, preserving adjacent neurovascular structures, and the joint capsule was repaired (Fig. 2). The procedure was performed by a hand specialist and lasted 1 hour.

**Figure 3.**
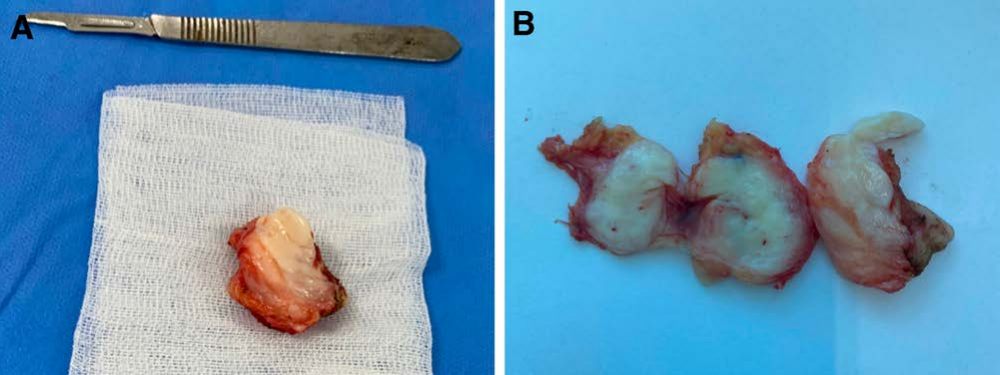
Gross findings of the tumor. (A) A well-circumscribed, firm, and lobulated tumor. (B) A homogeneous, fibrous, and glistening cut surface, occasionally displaying whorled or trabeculated areas due to dense collagenous tissue.

**Figure 4.**
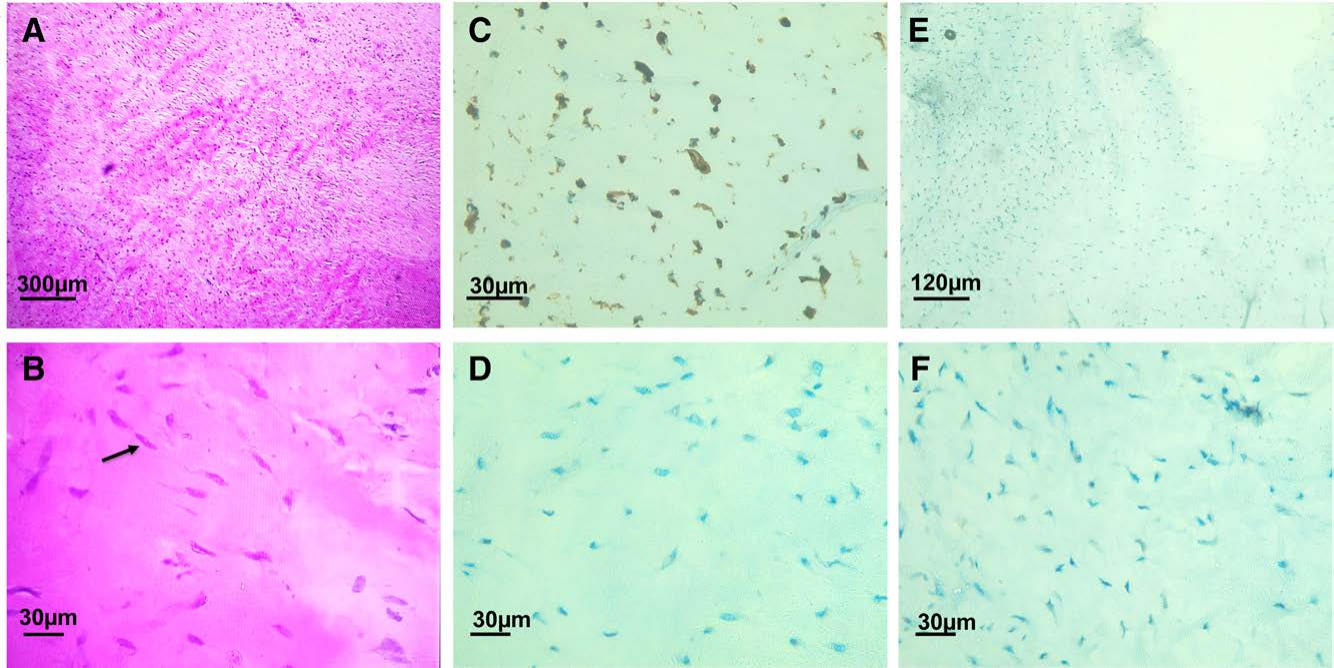
Histopathological examination. (A) Hematoxylin and eosin (H&E) stain, 40×: bundles of wavy spindle cells in a collagenous stroma. (B) H&E stain, 400×: low cellularity with uniform spindle cells (arrow) in a collagenous stroma. (C) Immunohistochemistry (IHC), 400×: tumor cells positive for vimentin. (D) IHC, 400×: tumor cells negative for S-100. (E) IHC, 100×: tumor cells negative for CD68. (F) IHC, 400×: tumor cells negative for SMA. H&E = hematoxylin and eosin, IHC = immunohistochemistry, SMA = smooth muscle actin.

Gross examination revealed a smooth, multilobulated, white mass without myxoid changes or necrosis (Fig. 3). Microscopic evaluation showed dense, hypocellular fibrous tissue with spindle-shaped fibroblasts within a collagenous stroma. No mitotic activity, atypia, or necrosis was observed. Immunohistochemical staining was positive for vimentin but negative for S-100, CD68, and smooth muscle actin, excluding neurogenic and vascular tumors (Fig. 4). While additional markers such as CD34 and molecular testing for FUS-CREB3L2 fusion were not performed due to limited resources, this diagnostic limitation has been acknowledged in Section 3. Based on the histological and immunohistochemical profile, a definitive diagnosis of FTS was established. The surgical approach remained unchanged.

The postoperative period was uneventful. The patient showed significant improvement in QuickDASH and visual analog scale scores after 1 week, with 21 and 2, respectively. At 6-month follow-up, she experienced complete resolution of symptoms, with no signs of recurrence. The patient expressed satisfaction with the treatment outcome, particularly the relief from pain and numbness. She reported significant improvement in hand function and quality of life, allowing her to resume daily activities without discomfort.

## 3. Discussion

This case underscores the need to differentiate FTS from other soft tissue tumors, particularly in cases presenting with median nerve compression. The patient presented with median neuropathy symptoms. The key differential diagnosis was whether the tumor originated from the nerve or was an external compressing mass. Peripheral nerve sheath tumors, such as schwannomas or neurofibromas, arise from nerve fascicles, causing nerve displacement or infiltration. These tumors typically remain asymptomatic until reaching a size of 25 to 30 mm in diameter, at which point they begin compressing nerve fibers.^[5]^ GCTTS is another critical differential diagnosis.^[6]^

MRI and intraoperative findings help distinguish between these tumors. Neurogenic tumors typically exhibit a “target sign” on MRI, while GCTTS often appear isointense to muscle on T1-weighted images and hyperintense on T2-weighted images due to increased vascularity.^[5,6]^ FTS, by contrast, is typically hypointense on both T1- and T2-weighted images due to its dense collagenous nature and shows little to no enhancement.^[6]^ In our case, the tumor appeared heterogeneously hyperintense on T2-weighted images – an atypical feature possibly reflecting lower collagen density, vascularity, or subtle myxoid change. This discrepancy emphasizes the variability in FTS imaging. Although histopathology and immunohistochemistry (positive for vimentin; negative for S-100, CD68, smooth muscle actin) confirmed the diagnosis, the absence of CD34 staining or FUS-CREB3L2 fusion testing is a limitation we acknowledge. It highlights the value of comprehensive diagnostic evaluation in atypical presentations.

FTS is a rare cause of carpal tunnel syndrome.^[3]^ The tumor can compress the median nerve proximally, distally, or at the level of the carpal tunnel. A simple release of the flexor retinaculum is insufficient in cases of direct compression. Anselmo et al described a similar case, though with a smaller tumor.^[7]^ Intra-articular involvement of FTS is infrequent. Only 2 previous cases of intra-articular FTS in the wrist have been reported, one in the distal radioulnar joint and another in the radiocarpal joint.^[8,9]^ Joint involvement raises concerns about synovial irritation, mechanical restriction, and potential joint damage, necessitating careful surgical excision to preserve joint integrity.^[9,10]^ FTS is best treated via marginal excision while preserving adjacent structures. Incomplete excision increases the risk of recurrence, which has been reported in up to 24% of cases.^[1]^ In this case, surgical excision of the tumor effectively alleviated the symptoms and also allowed for histological confirmation of the diagnosis. Even with careful surgery, there’s still a small risk of nerve damage, especially if the tumor is located close to the nerve. Our patient underwent successful excision without recurrence, but long-term follow-up remains essential. Therefore, extended clinical and imaging follow-up is warranted to ensure long-term disease control and timely management of any recurrence.

In conclusion, this case highlights the rare occurrence of FTS extending into the radiocarpal joint with median nerve compression. MRI plays a crucial role in early diagnosis and surgical planning, particularly in identifying intra-articular involvement. However, histopathological examination remains the gold standard for definitive diagnosis. Complete surgical excision is essential to prevent recurrence, requiring meticulous preservation of joint integrity and nerve function to optimize patient outcomes.

Supplemental Digital Content “Supplementary Figure 1” is available for this article (https://links.lww.com/MD/P596).

## Author contributions

**Conceptualization:** Nhat Tien Tran, Thuy Tram Ngo, Bao Song Nguyen Tran, Hong Phuc Le, Nghi Thanh Nhan Le.

**Data curation:** Nhat Tien Tran, Bao Song Nguyen Tran.

**Formal analysis:** Nhat Tien Tran, Thuy Tram Ngo, Bao Song Nguyen Tran, Nghi Thanh Nhan Le.

**Funding acquisition:** Nghi Thanh Nhan Le.

**Investigation:** Nhat Tien Tran, Bao Song Nguyen Tran, Hong Phuc Le.

**Methodology:** Nhat Tien Tran, Hong Phuc Le.

**Project administration:** Nghi Thanh Nhan Le.

**Resources:** Nhat Tien Tran.

**Supervision:** Nhat Tien Tran, Nghi Thanh Nhan Le.

**Validation:** Nhat Tien Tran, Thuy Tram Ngo, Nghi Thanh Nhan Le.

**Visualization:** Nhat Tien Tran, Thuy Tram Ngo, Bao Song Nguyen Tran, Hong Phuc Le.

**Writing – original draft:** Nhat Tien Tran, Thuy Tram Ngo.

**Writing – review & editing:** Nhat Tien Tran.

## Supplementary Material


