# Supplementary figures and images for: Fibroma of tendon sheath extending to the radiocarpal joint with median nerve compression: A case report
Source: Medicine (Baltimore). 2025 Aug 1;104(31):e43735. doi: 10.1097/MD.0000000000043735 (PMC12324010; doi:10.1097/MD.0000000000043735)

Supplementary Figure 1 Timeline:


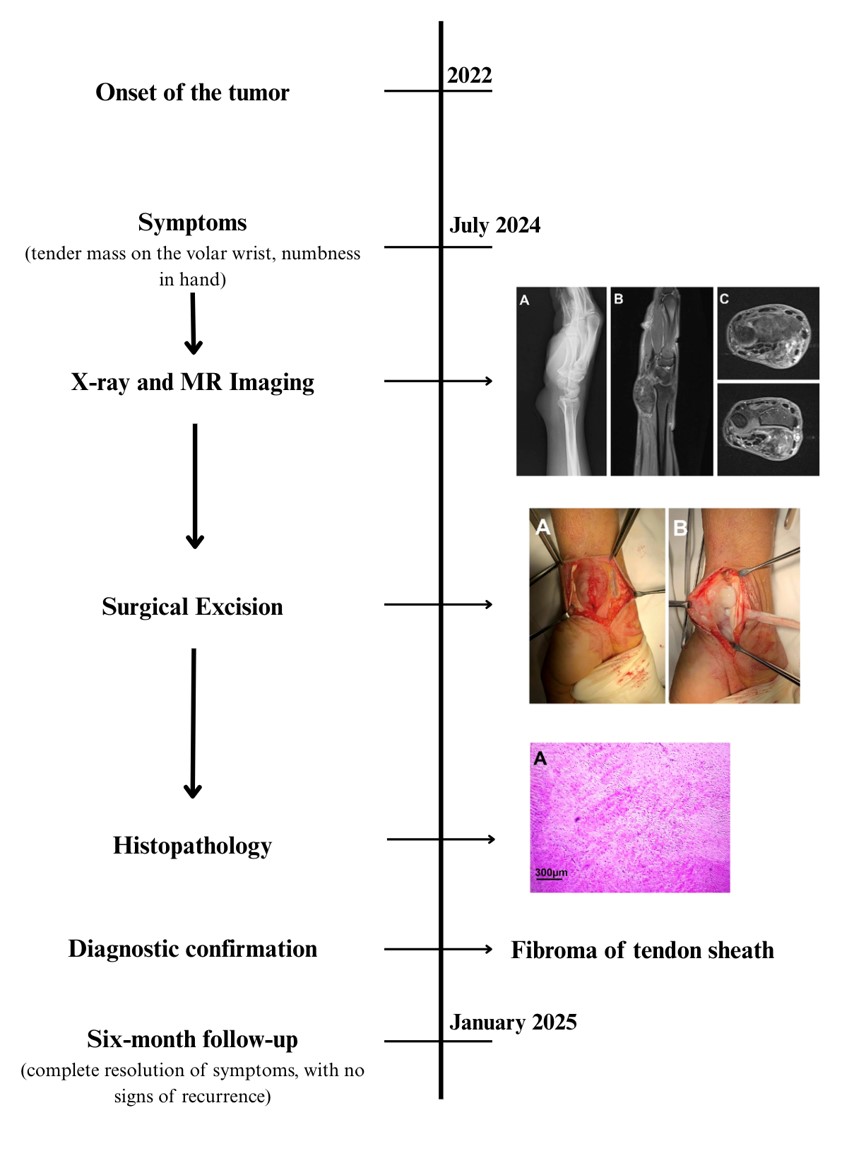

Supplement: Supplementary file 1 [file medi-104-e43735-s001.docx]
